# The King's Fund and Hospitals with Medical Schools

**Published:** 1911-04-01

**Authors:** 


### The Hospital

A Journal of

The Medical Sciences and Hospital Administration.

New Series. No. 217, Vol IX. [No. 1289, Vol. L.] SATURDAY, APRIL 1, 1911

### The King's Fund and Hospitals with Medical Schools.

In our issue of December 17 last we published an article dealing with the correspondence which had passed between the Metropolitan Radical Federation and the honorary secretaries of the King's Fund. In that correspondence the Metropolitan Radical Federation alleged that the King's Fund, in making a grant to St. George's Hospital in 1909, had condoned the action of St. George's Hospital in openly flouting the directions of its own distinguished Committee of Inquiry, known as Sir Edward Fry's Commission. This charge, as we showed at the time, was based on a misapprehension, for the intention of the Council of the King's Fund is, and always has been, to uphold the findings of Sir Edward Fry's Commission in the matter both as to the letter and the spirit. In fact, on the recommendation of the Distribution Committee, the grant to St. George's Hospital was retained until the King's Fund was satisfied that the financial relations between the hospital and school are in accordance with the principles intimated by the Fund in March 1909, and that the general account of St. George's Hospital in respect of 1909, as well as of 1910, is secure from any payment on account of medical education.

That the question is surrounded by difficulties anyone will perceive at once who carefully reads the report of the Distribution Committee on the suspended grant to St. George's Hospital, which appears in another column. This report displays the care and ability with which the Distribution Committee of this Fund discharges the very responsible and highly technical duties which devolve upon it. It covers a large number of technical points which are dealt with clearly and impartially, and must convince the Metropolitan Radical Federation, and all who are interested in this question, that the strictest impartiality and the fullest justice is meted out to all hospitals receiving grants, and especially those which are concerned in medical education. The Distribution Committee's report shows that the payments by St. George's Hospital in 1909, in respect of the specific work referred to, includes salaries, cash payments, and rent, making a total of £1,875. In making its inquiries the Committee carefully ascertained the cost of similar work at other hospitals, rightly holding that if the cost to St. George's of the laboratories is largely in excess proportionately to the cost of other comparable hospitals, the difference required investigation. Pursuing its inquiries upon this principle, it arrived at the conclusion that at St. George's the sum paid by the hospital appears from the returns submitted to represent about four-fifths of the total cost of laboratory investigations, while the school pays about one-fifth. The Committee found from the figures supplied by the remaining institutions that the cost of these investigations at St. George's Hospital worked out at about two and a half times the average if St. George's was included, or slightly over two and three-quarter times if St. George's was excluded.

The Committee had next to consider whether these differences of cost in this connection were due to (1) the relative amount of the total expenditure on the laboratory work; or (2) the differences in the method of its apportionment as between hospital and medical school. After making full allowance for certain changes which have taken place at St. George's Hospital medical school since Sir Edward Fry's Committee reported, it came to the conclusion to base its decision upon the broad ground of comparative cost, because there is no reason to suppose that the work done at St. George's differs markedly in quality or quantity from that done at other London hospitals of equal repute. In the result the Distribution Committee has come to the conclusion that the King's Fund ought to mark its disapproval of expenditure so far in excess of the average, whether such excess arises from extravagance, from a contribution to the medical school, or from the clinical laboratories being designed not only for hospital requirements, but also for purposes of research.

21360

The Committee therefore finds that the St. George's Hospital School should repay to the hospital in respect to the year 1909 (and if necessary 1910) the difference between the sum of £1,000 and the charge borne by the hospital in respect to all specific work done for the hospital patients, which amounted in 1909 to £1,875.

The accounts of St. George's Hospital for the year 1909 will therefore have to be adjusted by the repayment by the Medical School of £875 to the hospital for that year. It is satisfactory to note that in arriving at this conclusion the Distribution Committee desires to state that in its opinion the certificate given to the Fund by the Treasurer of St. George's Hospital, to the effect that no payments have been made to or on account of the Medical School for the purposes of medical education, was given in absolute good faith. The matter, as we have said, is of much complexity, and we endorse the Committee's opinion that different conclusions as to the proper apportionment of this expenditure have been reached by different persons whose bona-fides are equally unquestioned. The Distribution Committee deserves the warm acknowledgments of all friends of the voluntary hospitals, and of the public generally, and its report, which was adopted by the Council of the King's Fund on March 28, will, we hope, put an end to a controversy which will have no basis to rest upon so long as the present policy of the King's Fund Council in upholding the findings of Sir Edward Fry's Commission in this matter is adhered to.

